# Comparative analysis of feeding probiotic and exogenous buffer on growth performance, nutrient utilization, blood biochemistry, rumen fermentation pattern and carcass characteristics of crossbred lambs reared under feedlot

**DOI:** 10.3389/fvets.2026.1964601

**Published:** 2026-09-30

**Authors:** Qazi Shehriyar Sahib, Haidar Ali Ahmed, Ifla Aafaq, Javid Farooq, Tahir Nazir, Zubair Ahmad Akhoon

**Affiliations:** 1 Division of Animal Nutrition, Faculty of Veterinary Sciences and Animal Husbandry, SKUAST-Kashmir, Srinagar, Jammu and Kashmir, India; 2 Division of Animal Nutrition, Faculty of Veterinary Sciences and Animal Husbandry, Jammu, Jammu and Kashmir, India; 3 Division of Livestock Products Technology, Faculty of Veterinary Sciences and Animal Husbandry, SKUAST-Kashmir, Srinagar, Jammu and Kashmir, India; 4 Division of Clinical Veterinary Medicine, Ethics and Jurisprudence, Faculty of Veterinary Sciences and Animal Husbandry, SKUAST-Kashmir, Srinagar, Jammu and Kashmir, India

**Keywords:** exogenous buffer, feedlot lambs, nutrient utilization, performance, probiotic

## Abstract

**Background:**

Feedlot nutrition is formulated to maximize growth rate and feed efficiency for high-quality meat production; however, high-grain diets can disrupt rumen function and cause digestive disorders, the inclusion of non-nutritive feed additives helps counter these effects, thereby enhancing growth performance and overall feed efficiency.

**Methods:**

The present study was carried out to delineate the effect of feeding *Saccharomyces cerevisiae* strain 1,026 as probiotic and sodium bicarbonate as exogenous buffer on performance of feedlot crossbred lambs. Fifteen crossbred lambs were selected and divided equally into three treatment groups *viz*., FT, FT-P and FT-SB. In FT group, animals were fed feedlot diet only; while in FT-P and FT-SB groups, *Saccharomyces cerevisiae* and sodium bicarbonate were offered as feed additives, respectively. A 74-days feeding trial was conducted, including 10-days metabolism trial at the end.

**Results:**

No effect (*p* > 0.05) was observed in dry matter intake (DMI), body weight indices, feed conversion ratio (FCR), digestibility coefficients of nutrients and haemato-biochemical parameters. Rumen pH was significantly (*p* < 0.05) higher in FT-P group than FT group. Dressing percentage showed significantly (*p* < 0.05) higher values in FT-P group.

**Conclusion:**

The present experiment validates that feeding of *Saccharomyces cerevisiae* had more beneficial effect than sodium bicarbonate feeding on the performance of feedlot lambs.

## Introduction

Farmers who are involved in the steady supply of lambs to the market try to fulfill the standard for weight and fat score through managerial techniques such as “feedlotting” (1). It enables producers to maintain production when pasture is scarce, achieve rapid growth when feed prices are low, generate cash flow, and provide flexibility in finishing lambs throughout the year (2). The FTs bring various benefits in finishing lambs, such as greater weight gain, lower age at slaughter, higher carcass yield, lower mortality, and lower worm infestations (1). The average FT period lasts 60–80 days, during which the animal will typically acquire 15–20 kg. In this system, normally ADG is above 250 g, however, 400 g/day or above can also be achieved by animals that have a higher weight gain potential, which comes from genetic traits (3). Feeding a lamb with poor conformation intensively under FTs adds value to the carcass by improving muscular tissue growth and fat deposition, resulting in a more appealing meat product (4).

Under the FT system of rearing, digestive problems account for between 14% and 42% of cattle deaths (5). Clinical rumen acidosis is the result of ruminants who are not acclimated to a high-concentrate diet consuming excessive amounts of rapidly fermentable carbohydrates, which allows organic acids to build up in the rumen and cause a rumen fluid pH of less than 5.2 (5). The volatile fatty acids (VFAs) and lactate are produced in excess when fast-fermenting carbohydrates are fed in excess, a condition known as “grain overload” (6). Lactate production by *Streptococcus bovis* decreases with decreasing pH, and *Streptococcus bovis* growth slows (7). At this stage, *Lactobacilli* take over as the predominant rumen micro-organisms, significantly lowering the ruminal pH. Rumen microbes produce a wide range of additional substances besides lactate that can harm other organs. These deleterious compounds include endotoxins and histamine (5). Rumen fluid analysis is one of the most effective diagnostic techniques for suspected rumen acidosis. Animals suffering from acidosis may have rumen fluid of watery consistency, milk-gray color, and putrid odor. In any case, rumen acidosis is diagnosed when the pH of the rumen fluid falls below 5.5 (8).

Proper utilization of feed additives such as limestone, bicarbonate, and ionophore antimicrobials may lessen the incidence and severity of disease (8). The use of non-nutritive additives, such as buffers, ionophores, malate, and direct-fed microorganisms, may lessen ruminal acidity and associated health issues (9). Incorporating *S. cerevisiae* (as a probiotic) into ruminant diets causes the rumen environment to absorb and consume oxygen, while also releasing certain vital enzymes, vitamins, and other nutrients (10). These factors may significantly affect the survival and activity of microorganisms in the ruminal environment. According to Calsamiglia et al. (11), *S. cerevisiae* may boost the population of *Megasphaera elsdenii* (Gram-negative bacteria). In turn, these bacteria prevent the sharp decrease in ruminal pH by consuming lactic acid as a result of lactic acid accumulation (12). When compared to pharmaceuticals, the usage of yeast and bioactive substances can effectively reduce the inflammation brought on by acidosis (13).

Sodium bicarbonate is frequently added to the rumen to buffer its pH, but because of its p*K*a of 6.25, its buffering efficacy is diminished when rumen pH is acidic (5.5–5.8) (14). Furthermore, sodium bicarbonate has a quick but short-lived buffering effect and quickly dissolves into rumen fluid. Because of this, a high amount is required to buffer the rumen pH of cattle fed high-energy diets (0.7–1% of DM) (15). A high amount of sodium bicarbonate, when given with diets rich in phosphorus, increases the risk of developing urolithiasis (16).

The present experiment was designed to compare the effect of feeding *S. cerevisiae* and sodium bicarbonate as feed additives on the growth performance, nutrient digestibility, blood biochemistry, rumen fermentation, and carcass characteristics of crossbred finishing lambs reared under FT system.

## Materials and methods

### Location of the study

The present study has been conducted on crossbred male lambs maintained at Mountain Research Centre for Sheep and Goat (MRCS&G), Shuhama, SKUAST-K (34.1886°N; 74.8286°E), situated at an altitude of 5,212 ft above sea level. The environmental temperature of the region ranges from −7 °C during winter (December–February) to 35 °C during summer (June–August), with an average annual rainfall of 744 mm and a mean annual temperature of 13.4 °C.

### Animal source and housing

Fifteen healthy crossbred male lambs (12 ± 02 months of age; 20.49 ± 0.68 kg body weight) were selected for the experiment. The lambs for the experiment were procured from the sheep sub-unit of MRCS&G, Shuhama, SKUAST-K. Initially, an adaptation period (14 days) was given to the animals under trial for their acclimatization in the experimental shed and to adapt to the FT diet before the start of the experiment. After 14 days, the animals were weighed before feeding and watering in the morning for two consecutive days, and were distributed into three equal groups to have uniform body weight. Throughout the experimental period, all the animals were kept under uniform management conditions in individual housing pens with the provision of individual feeding within well-ventilated sheds.

### Experimental design and diets

The animals were treated during the experiment in accordance with the Institutional Animal Ethics Committee of SKUAST-Kashmir's approved trial protocol regulations (Registration no.: 1809/GO/ReBiS/ReL/15/CPCSEA). All the experiments were performed in accordance with relevant guidelines and regulations. The design for the experiment followed was a completely randomized design (CRD). The three dietary treatments in the experiment were control (FT), treatment-1 (FT-P), and treatment-2 (FT-SB). Table 1 shows the concentrate mixture's ingredient composition. FT diets consisting of 20% roughage and 80% concentrate were provided to the experimental groups (as per ICAR, 2013 recommendations) (17). The FT group did not receive any feed additive; however, in the FT-P group *S. cerevisiae* strain 1026 (live yeast probiotic culture) was fed @1 g/animal/day as a feed additive, and in the FT-SB group, sodium bicarbonate was added @1% of total DMI of the animal. Total mixed ration (TMR) was made by combining roughage and concentrate mixture, and each experimental animal was offered an equal portion of TMR twice a day, in the morning (8:00 h) and evening (16:00 h). *S. cerevisiae* was provided after feeding to each experimental animal in the FT-P group, while the dose of sodium bicarbonate (1% of DMI) was calculated and added to the TMR. Clean drinking water was also provided ad-lib throughout the experimental period. The daily dry matter intake (DMI) was calculated by subtracting the residue (DM basis) left by the experimental animals each day from the feed offered (DM basis) during the 74-day study period.

**Table 1:** Composition of concentrate mixture and chemical composition of concentrate, roughage and TMR offered to experimental animals.

Composition of concentrate mixture	
Components, %	Content
Maize	54
Mustard oil cake	23
Wheat bran	12
Cowpea	8
Vitamin mineral premix for sheep*	2
Salt	1

Chemical composition of concentrate, roughage and TMR offered to experimental animals			
Attributes	Concentrate	Oat hay	TMR
Proximate composition			
DM	89.17	87.91	88.90
CP	18.96	6.56	16.48
EE	4.91	1.20	4.17
CF	5.65	34.15	11.35
NFE	65.75	52.18	63.04
TA	4.74	5.92	4.97
AIA	0.17	2.09	0.55
Fiber fractions			
NDF	19.40	62.80	28.08
ADF	9.90	41.10	16.14
HC	9.50	21.70	11.94
Cellulose	6.60	32.80	11.84
ADL	4.00	7.40	4.30
Minerals			
Calcium	0.31	0.54	0.35
Phosphorus	0.59	0.15	0.50

*Vitamin mineral premix for sheep: Vitamin A–7,00,000 I. U, Vitamin D_3_–70,000 I. U, Vitamin E–250 mg, Nicotinamide–1,000 mg, Co–200 mg, I–325 mg, Fe–1,500 mg, Mg–6,000 mg, Mn–1,500 mg, K–100 mg, Na–5.9 mg, S–0.72%, Zn–15 gm, Ca–25% and P–12.75%.

DM, dry matter; CP, crude protein; EE, ether extract; CF, crude fiber; NFE, nitrogen free extract; TA, total ash; AIA, acid insoluble ash, NDF, neutral detergent fiber; ADF, acid detergent fiber; HC, hemicellulose, ADL, acid detergent lignin.

### Procurement and chemical analysis of feed ingredients

Feed ingredients utilized in the formulation of experimental rations and sodium bicarbonate were procured from the local market, while the *S. cerevisiae* strain 1026 (Tradename: Yea-Sacc^®^) was procured from Equine Nutrients, Bangalore, India. The experimental ration and feed ingredients were examined for their proximate principles (CP, CF, EE, NFE, and TA) by AOAC (18), fiber fractions (NDF, ADF, Hemicellulose, Cellulose, and Lignin) by Van Soest et al. (19), and two major minerals, namely, calcium and phosphorus, by Talapatra et al. (20). Table 1 shows the chemical composition of concentrate, roughage, and TMR offered to the experimental animals.

### Growth parameters and metabolic studies

To assess the body weight changes, the body weights of experimental animals were recorded at the initiation of the experiment and then at fortnightly intervals using an electronic weighing scale. For the growth study, the body weight of each experimental animal was recorded before feeding and watering in the morning. To assess the actual effect of diet, total body weight gain, fortnightly body weight gain, ADG (g/d), and FCR were calculated. The FCR was calculated by the following formula:


$$\mathrm{FCR}=\frac{\text{Total Feed intake}\;(\mathrm{kg})}{\text{Total gain in body weight}\;(\mathrm{kg})}$$

Following the feeding trial, a 10-day metabolic trial (including 3 days for adaptation) was carried out with all the animals in the experiment. To assess digestibility, nutritional plane, and nitrogen balance, a 10-day metabolic trial (including a 3-day adaptation phase) was carried out from the 65th to 74th day of the experiment using metabolic cages measuring 2 m × 0.55 m in total surface area. Representative samples of the offered feeds and their corresponding residues were collected, oven-dried at 100°C for 24 h, and analyzed for proximate composition by AOAC (18). Urine and feces excreted by the experimental animals during the metabolism trial were collected. The representative samples of urine and feces were preserved in toluene and 25% H₂SO₄, respectively, for subsequent laboratory analysis.

### Hemato-biochemical studies

Blood samples were collected from the jugular vein of each experimental animal using 18G needles under strict aseptic precautions. Sampling was carried out at the beginning of the experiment and subsequently at 30-day intervals, in the morning prior to feeding and watering. The blood was then collected in Na_2_EDTA vials (anticoagulant). Hematological studies, namely, Hb and packed cell volume (PCV), were performed using Sahli’s method and the Microhematocrit method, respectively, soon after blood collection. For estimation of blood glucose, liver function tests (alanine transaminase (ALT), aspartate aminotransferase (AST), and alkaline phosphatase (ALP)), and kidney function tests (BUN and serum creatinine), serum was obtained from blood by applying conventional separation techniques, and further estimation was done by using commercial diagnostic kits procured from Span Diagnostics Limited, Surat, Gujarat, India.

### Rumen fermentation parameters

The rumen liquor samples were collected from the experimental lambs on the initial and final day of the trial in the morning (before feeding), with the help of a perforated stomach tube (50 mm), introduced through the wooden mouth gag into the rumen. The pH of the rumen fluid was determined using a portable digital pH meter (pen type) immediately after collection of rumen liquor. The estimation of total volatile fatty acids (TVFAs) was conducted by the protocol proposed by John et al. (21). The estimation of ammonia nitrogen (NH_3_-N) and total nitrogen was conducted by distillation of strained rumen liquor in a Kjeltec automatic distillation system (Pelican equipment). Trichloroacetic acid (TCA) precipitable nitrogen was estimated as per the method of Cline et al. (22), while the non-protein nitrogen (NPN) was calculated by subtracting the values of TCA-precipitable nitrogen from total nitrogen.

### Carcass characteristics

The experimental animals were kept off feed overnight, and water was withdrawn 3–4 h prior to slaughter. The experimental animals were weighed before slaughter. The experimental animals were slaughtered by the *Halal* method and were left to bleed until all the blood was drained out. The parameters recorded were bleeding time, bleeding percentage, dressing percentage, and loin eye area. The bleeding percentage and dressing percentage are calculated by the formula mentioned below:


$$\text{Bleeding percentage}=\frac{\text{Live weight}-\text{Weight after bleeding}}{\text{Live weight}}x\;100$$

$$\text{Dressing percentage}=\frac{\text{Dressed weight}}{\mathrm{Pre}-\text{slaughter live weight}}x\;100$$

Different major cuts (neck, shoulder, rack, loin, and legs) and minor cuts (breast, flank, and shank) of the carcass were weighed individually. The weight of cut-up parts (%) is calculated by the formula:


$$\%\mathrm{Cut}-\mathrm{up}\;\text{part weight}=\frac{\text{Weight of individual}\;\mathrm{cut}}{\text{Dressed carcass weight}}x\;100$$

### Statistical analysis

The data of feed intake, nutrient digestibility, growth performance, blood biochemistry, rumen fermentation, and carcass characteristics are presented as means with SEM and *P*-value. The differences between treatment groups were analyzed by one-way analysis of variance (ANOVA) using SPSS version 23.0 for macOS. The statistical equation used was.


$$A_{\mathrm{ij}}=\mu +B_{i}+e_{\mathrm{ij}}$$

where *A_ij_* represents the parameter under investigation; *μ* denotes the overall mean; *B_i_* indicates the effect of treatments on the parameter being analyzed; and *e_ij_* refers to the experimental error associated with the *ij*th observation. The effects were regarded as statistically significant at a *P*-value of < 0.05.

## Results

### Feed consumption and gain in body weight

Results of DMI (g/d) did not show any significant difference (*P* > 0.05) between the treatment groups (Table 2). The body weight indices, namely, total gain in body weight (kg), fortnight body weight gain (kg), ADG (kg), and FCR, showed no significant effect (*P* > 0.05) between the treatments (Table 2).

**Table 2:** Feed consumption and body weight gain of experimental animals under different feeding regimen.

Parameter	Groups			SEM	*p*-value
	FT	FT-P	FT-SB		
DMI (g/d)	908.85	898.93	940.04	34.26	0.223
Initial BW (kg)	20.52	20.49	20.46	0.68	0.999
Final BW (kg)	29.94	31.67	31.68	0.98	0.743
Total gain in BW (kg)	9.42	11.18	11.22	0.66	0.478
ADG (g/d)	127.29	151.08	151.62	8.80	0.478
FCR	7.14	5.95	6.20	0.33	0.328

FT, feedlot; FT-SC, feedlot with *Saccharomyces cerevisiae* as feed additive; FT-SB, feedlot with sodium bicarbonate as feed additive.

DMI, dry matter intake; BW, body weight; ADG, average daily gain; FCR, feed conversion ratio.

SEM, standard error mean.

### Digestibility coefficients, nutritive value of experimental ration, intake of digestible nutrients, and nitrogen utilization

No significant effect (*P* > 0.05) was observed in the apparent digestibilities of gross nutrients, namely, DM, OM, CP, EE, CF, NFE, NDF, ADF, cellulose, and HC (Table 3). The data revealed non-significant difference (*P* > 0.05) in terms of DCP (%) and TDN (%) values between the experimental groups (Table 3). Non-significant differences (*P* > 0.05) were observed in terms of DDMI (kg/d), DCPI (g/d), and TDNI (kg/d) values in the statistical analysis (Table 3).

**Table 3:** Average digestibility coefficients, nutritive value of experimental rations, intake of digestible nutrients and nitrogen utilization of experimental animals under different feeding regimen.

Parameter	Groups			SEM	*P*-value
	FT	FT-P	FT-SB		
Digestibility coefficient of gross nutrients					
DM	61.71	64.44	63.33	1.93	0.870
OM	69.91	72.71	72.08	1.11	0.606
CP	68.49	76.93	74.08	2.79	0.500
EE	59.46	74.70	74.39	5.62	0.493
CF	61.96	71.18	67.73	3.42	0.586
NFE	71.48	73.55	72.76	2.02	0.930
NDF	43.64	50.62	50.01	4.33	0.801
ADF	35.54	41.36	45.68	5.88	0.812
Cellulose	59.28	66.97	64.09	3.31	0.677
HC	54.60	63.14	55.85	3.08	0.518
Nutritive value of experimental rations					
DCP (%)	11.29	12.68	12.21	0.46	0.500
TDN (%)	68.96	74.13	72.75	2.48	0.719
Intake of digestible nutrients					
DDMI (kg/d)	0.83	0.74	0.85	0.05	0.651
DCPI (g/d)	137.49	128.12	144.04	8.97	0.801
TDNI (kg/d)	0.83	0.60	0.86	0.06	0.257
Nitrogen utilization					
Nitrogen intake (g/d)	30.78	26.79	31.24	1.40	0.402
Faecal nitrogen (g/d)	12.27^c^	7.92^a^	10.48^b^	0.55	<0.05
Urinary nitrogen (g/d)	5.65^a^	6.26^ab^	7.52^b^	0.35	0.079
Nitrogen balance (g/d)	12.86	12.61	13.24	1.40	0.986
Nitrogen retention (%)	40.09	44.42	41.93	3.51	0.900
Nitrogen absorbed (%)	59.21^a^	69.32^b^	66.32^ab^	2.02	0.102

^abc^means bearing different superscripts within a row differ significantly at 5% level (*p* < 0.05).

FT, feedlot; FT-SC, feedlot with *Saccharomyces cerevisiae* as feed additive; FT-SB, feedlot with sodium bicarbonate as feed additive.

DM, dry matter; OM, organic matter, CP, crude protein; EE, ether extract; CF, crude fiber; NFE, nitrogen free extract, NDF, neutral detergent fiber; ADF, acid detergent fiber; HC, hemicellulose; DCP, digestible crude protein; TDN, total digestible nutrients; DDMI, digestible dry matter intake; DCPI, digestible crude protein intake; TDNI, total digestible nutrients intake.

In statistical relations, the data related to nitrogen utilization showed significant differences (*P* < 0.05) among treatments in terms of fecal nitrogen (g/d), urinary nitrogen (g/d), and nitrogen absorbed (%) (Table 3). On comparing means of nitrogen absorbed (%), higher values were observed in the *S. cerevisiae*-supplemented FT group, followed by the sodium bicarbonate-supplemented group, and the least in the FT group without feed additive (Table 3).

### Hemato-biochemical studies

The results of various hemato-biochemical parameters, namely, Hb, PCV, blood glucose, ALT, AST, ALP, BUN, and creatinine are presented in Table 4. The values of Hb, PCV, and blood glucose revealed non-significant (*P* > 0.05) difference when subjected to statistical analysis. On comparing means of liver enzymes, namely, ALT, AST, and ALP, also showed no significant difference (*P* > 0.05) was observed between treatments. The kidney function tests, namely, BUN and creatinine showed a non-significant (*P* > 0.05) effect of incorporating *S. cerevisiae* and sodium bicarbonate in the FT diets.

**Table 4:** Haemato-biochemistry of experimental animals under different feeding regimen.

Parameter	Groups			SEM	*P*-value
	FT	FT-P	FT-SB		
Haemoglobin (g/dl)	8.48	9.66	8.89	0.33	0.372
Packed cell volume (%)	25.43	28.97	26.66	1.00	0.372
Blood glucose (mg/dl)	63.15	65.30	68.00	2.02	0.653
ALT (IU/L)	16.05	15.89	15.94	0.70	0.996
AST (IU/L)	98.89	97.15	96.10	2.47	0.911
ALP (IU/L)	121.66	117.07	119.72	1.82	0.623
Blood urea nitrogen (mg/dl)	16.49	15.99	15.04	0.54	0.582
Serum creatinine (mg/dl)	1.49	1.54	1.51	0.03	0.825

FT, feedlot; FT-SC, feedlot with *Saccharomyces cerevisiae* as feed additive; FT-SB, feedlot with sodium bicarbonate as feed additive.

ALT, alanine transaminase; AST, aspartate aminotransferase, ALP, alkaline phosphatase.

SEM, standard error mean.

### Rumen fermentation parameters

The results of rumen fermentation parameters, namely, rumen pH, TVFA, and nitrogen fractions (total nitrogen, ammonia nitrogen, TCA precipitable nitrogen, and NPN), are presented in Table 5. The results of rumen pH and TVFA revealed significant differences (*P* < 0.05) between treatments, showing statistically higher values in the *S. cerevisiae*-supplemented group, followed by the sodium bicarbonate-supplemented group and then the FT group without feed additive. The mean values of total nitrogen, ammonia nitrogen, TCA precipitable nitrogen, and NPN showed no significant difference (*P* > 0.05) between the treatment groups.

**Table 5:** Rumen fermentation of experimental animals under different feeding regimen.

Parameter	Groups			SEM	*P*-value
	FT	FT-P	FT-SB		
Rumen pH	6.10^a^	6.73^b^	6.70^b^	0.11	0.006
TVFA (meq /dl)	9.50	9.85	9.33	0.09	0.074
Total nitrogen (mg/dl)	144.67	158.67	157.69	3.86	0.258
Ammonia nitrogen (mg/dl)	16.24	14.93	16.80	0.87	0.727
TCA precipitable nitrogen (mg/dl)	93.33	106.40	105.47	4.93	0.548
NPN (mg/dl)	51.33	52.27	53.20	3.48	0.982

^abc^means bearing different superscripts within a row differ significantly at 5% level (*p* < 0.05).

FT, feedlot; FT-SC, feedlot with *Saccharomyces cerevisiae* as feed additive; FT-SB, feedlot with sodium bicarbonate as feed additive.

TVFA, total volatile fatty acids; TCA, trichloroacetic acid; NPN, non-protein nitrogen.

SEM, standard error mean.

### Carcass characteristics

The average values of carcass traits of the experimental animals are presented in Table 6. The means of bleeding percentage and dressing percentage showed a significant difference (*P* < 0.05) between treatments. The statistically higher bleeding percentage values were observed in the FT group without feed additives, followed by the *S. cerevisiae*-supplemented group, and the lowest in the sodium bicarbonate-supplemented group. The dressing percentage was higher in the *S. cerevisiae*-supplemented group and lowest in the sodium bicarbonate-supplemented group. Among various major and minor carcass cuts, non-significant difference (*P* > 0.05) was observed in the statistical analysis.

**Table 6:** Carcass characteristics of experimental animals under different feeding regimen.

Parameter		Groups			SEM	*P*-value
		FT	FT-P	FT-SB		
Bleeding time (min)		4.65	3.92	4.19	0.13	0.067
Bleeding percentage		4.88^b^	4.26^a^	4.17^a^	0.12	0.004
Dressing percentage		44.89^b^	45.90^b^	42.66^a^	0.52	0.003
Loin eye area (inch^2^)		3.24	3.70	3.41	0.22	0.755
Weight of cut-up parts (%)						
Major cuts	Neck	4.75	4.23	4.02	0.23	0.461
	Shoulder	21.11	21.84	19.23	0.70	0.336
	Rack	6.29	5.82	5.28	0.20	0.119
	Loin	7.09	8.99	8.20	0.36	0.075
	Legs	31.19	31.59	32.53	0.38	0.403
Minor cuts	Breast	10.37	10.90	12.97	0.51	0.072
	Flank	6.11	7.42	6.80	0.26	0.127
	Shank	10.65	10.91	10.64	0.10	0.563

^abc^means bearing different superscripts within a row differ significantly at 5% level (*p* < 0.05).

FT, feedlot; FT-SC, feedlot with *Saccharomyces cerevisiae* as feed additive; FT-SB, feedlot with sodium bicarbonate as feed additive.

SEM, standard error mean.

## Discussion

### Feed consumption and gain in body weight

Predicting ration palatability depends on determining feed intake. The incorporation of *S. cerevisiae* as a feed additive in the present study did not show any significant difference in feed intake among treatment groups, and the study is in harmony with the results of Macedo et al. (23) and Sheikh et al. (24), who also reported no effect on feed intake following supplementation of yeast culture in the diets of experimental animals. In contrast, improvement in feed intake was observed by McDonald et al. (25) and Tripathi and Karim (26). Among all groups, the highest cumulative feed intake was found in the sodium bicarbonate-supplemented group and the lowest in the *S. cerevisiae-*supplemented group; however, the effect on DMI was non-significant. Similar results related to the use of sodium bicarbonate as a feed additive in the experimental ration were observed by Spears et al. (27) and Sahib et al. (15).

The results related to lambs fed on FT diets with feed additives revealed no significant difference among the treatment groups in terms of body weight gain. The results of supplementation of *S. cerevisiae* as a feed additive in the FT diets revealed numerically higher body weight gain, which showed harmony with the results of Sahoo et al. (28) and Sheikh et al. (24), who observed better growth and FCR in the yeast culture-supplemented group. Experimental animals fed FT diets with sodium bicarbonate as a feed additive showed similar body weight gain as reported by Fauchon et al. (29) and Mandebvu and Galbraith (30), who observed non-significant body weight changes in lambs fed diets supplemented with sodium bicarbonate.

### Digestibility coefficients, nutritive value of experimental ration, intake of digestible nutrients, and nitrogen utilization

In the present experiment, non-significant difference (*P* > 0.05) was found in the apparent digestibilities of DM, OM, CP, EE, CF, NFE, and fiber fractions (NDF, ADF, hemicellulose, and cellulose) between the treatment groups reared on FT diets with or without incorporation of feed additives. Although some nutrient digestion coefficients were numerically higher in the supplement groups, these differences were not statistically significant. Similar results in terms of supplementation of *S. cerevisiae* as a feed additive were found by Whitley et al. (31), Soren et al. (32), and Ovinge et al. (33). The results of supplementation with sodium bicarbonate as a feed additive were on similar lines with the results of Aguilera-Soto et al. (34), Farooq et al. (35), Martins et al. (36), Suman et al. (37), and Sahib et al. (15). As the researchers conveyed a non-significant difference in nutrient utilization of the sodium bicarbonate-fed group compared with other treatment groups.

### Hemato-biochemical studies

Neither *S. cerevisiae* nor sodium bicarbonate supplementation adversely affected the measured biochemical indicators of liver and kidney function. The results of the *S. cerevisiae*-supplemented group in terms of PCV values showed harmony with Sheikh et al. (24), who observed no effect on PCV with yeast supplementation. Hussein (38) found significant improvement in the values of Hb (%) in Najdi male lambs supplemented with prebiotics. Antunović et al. (39) and Ding et al. (40) observed no changes in the blood glucose levels in the diets of lambs containing probiotics. No significant difference (*P* > 0.05) was observed in ALT, AST, and ALP values of treatment groups compared with the control. The results of ALT and AST were similar to studies by El-Marakby (41) who observed no alterations serum concentrations. Contrary to the present findings, El-Nor and Kholif (42) and Butler (43) reported higher BUN values in response to probiotic supplementation, which may be associated with the incapacity of ruminal microflora to retain ammonia optimally.

### Rumen fermentation parameters

The outcome of the *S. cerevisiae*-supplemented group in terms of rumen pH showed harmony with Dawson and Tricarico (44), who demonstrated stimulation of rumen bacteria and enhancement of lactate and ammonia utilization following supplementation of feed with yeast, resulting in moderate pH. The study of Reis et al. (45) also concluded that the use of *S. cerevisiae* can be beneficial in the prevention and treatment of acute ruminal lactic acidosis in sheep, because it can effectively reduce the accumulation of lactic acid and thereby increase ruminal pH. In contrast, Hadjipanayiotou (46) reported significantly higher rumen pH (*P* < 0.01) at a 40 g/kg bicarbonate inclusion level over the control. González et al. (47), Apper-Bossard et al. (48), and Pérez‐Ruchel et al. (49), conveyed a similar increase in rumen pH with sodium bicarbonate supplementation. In this study, TVFA concentration was numerically higher in the *S. cerevisiae*-supplemented group, although the difference was not statistically significant. The study by Kawas et al. (50) reported that TVFA were not affected by the addition of sodium bicarbonate in the diets of finishing lambs. However, Garg et al. (51) reported that yeast supplementation significantly increased TVFA concentration compared with the control. In contrast to this study, Malik and Singh (52) observed higher values of ammonia nitrogen in all yeast-supplemented groups as compared to the control. Garg et al. (51) also reported that yeast supplementation significantly showed better rumen fermentation by decreasing ammonia nitrogen and increasing total nitrogen concentration in the rumen.

### Carcass characteristics

The literature related to the carcass characteristics of FT sheep is scanty. However, the lowest mean value of bleeding time (min) was recorded in the *S. cerevisiae*-supplemented treatment group and the highest in the FT group without feed additive; however, no significant difference (*P* > 0.05) was observed in bleeding time among the treatments. The overall mean values of dressing percentage showed significant differences (*P* < 0.05) between the treatment groups. The average values of dressing percentage were lowest for the sodium bicarbonate-supplemented group, and higher values were observed in the *S. cerevisiae*-supplemented group. The observation of Majdoub-Mathlouthi et al. (53) is in close agreement with the results of the present experiment; they observed that the high intake of concentrate did not affect the quality of carcass and meat quality (color, profile of fatty acids). The average values of cut-up parts (major and minor cuts) showed better results in the carcass characteristics of the *S. cerevisiae*-supplemented group as compared to that of the control group, which may be associated with better nutrient utilization and better growth rate. However, non-significant difference (*P* > 0.05) was found in the mean values of major and minor cuts between treatments. Results found on supplementation of probiotics show resemblance with the study of Pienaar et al. (54), who concluded that inclusion of *S. cerevisiae* in finishing diets for lambs had no effect (*P* > 0.05) on carcass characteristics of lambs.

## Conclusion

Supplementation of *S. cerevisiae* was more beneficial than sodium bicarbonate in FT lambs, particularly by improving ruminal pH, nitrogen utilization, and dressing percentage. However, the feed intake, growth rate, nutrient digestibility, and hemato-biochemical parameters remained unaffected. Thus, *S. cerevisiae* may be a promising feed additive for improving rumen stability and dressing percentage in FT lambs. Further studies using larger samples and more comprehensive measurements of ruminal fermentation and microbial activity are needed to elucidate the mechanisms underlying these responses.

## Data Availability

The original contributions presented in the study are included in the article/supplementary material, further inquiries can be directed to the corresponding author/s.
